# Risk Factors and Predictive Value of Depression and Anxiety in Cervical Cancer Patients

**DOI:** 10.3390/medicina58040507

**Published:** 2022-04-02

**Authors:** Suzana Tosic Golubovic, Iva Binic, Dane Krtinic, Vladimir Djordjevic, Irena Conic, Uros Gugleta, Marija Andjelkovic Apostolovic, Marko Stanojevic, Jelena Kostic

**Affiliations:** 1 Department for Psychiatry, Faculty of Medicine, University of Nis, 18000 Nis, Serbia; suzana.tosic.golubovic@medfak.ni.ac.rs (S.T.G.); vladimir_dj@mts.rs (V.D.); jelenakostic73@gmail.com (J.K.); 2 Clinic for Psychiatry, Clinical Center, 18000 Nis, Serbia; gugletauroszmaj@gmail.com; 3 Department for Pharmacology and Toxicology, Faculty of Medicine, University of Nis, 18000 Nis, Serbia; kdane86@gmail.com; 4 Clinic for Oncology, Department for Pharmacology and Toxicology, Clinical Center, 18000 Nis, Serbia; irenaconic@yahoo.com; 5 Center for Mental Health Protection, Clinical Center, 18000 Nis, Serbia; 6 Institute for Public Health, 18000 Nis, Serbia; drmari844@gmail.com; 7 Department for Medical Statistics and Informatics, Faculty of Medicine, University of Nis, 18000 Nis, Serbia; 8 Clinic for Gynecology and Obstetrics, Clinical Center, 18000 Nis, Serbia; dr.stanojevic@yahoo.com

**Keywords:** anxiety, depression, cervical cancer

## Abstract

*Background and Objectives*: Women with cervical cancer may experience depression or anxiety, influencing their quality of life and even their adherence to cancer treatments. This study aimed to explore and measure the levels of anxiety and depression in patients suffering from cervical cancer and to identify the possible predictors among known risk factors such as age, cancer stage, smoking status, number of partners, use of contraceptives, and annual gynecological visits. *Materials and Methods*: In total, 59 patients with cervical cancer were included. A consecutive sampling method was used to select participants in this research. Depression and anxiety were assessed using the Zung Anxiety Scale (SAS) and Zung Depression Scale (SDS). The subjects were divided into three groups, according to the stage of cancer. *Results*: Scores of depression and anxiety were increased in all recruited cervical cancer patients. A significant correlation was found between disease stage and the scores of depression (*p* = 0.002) and anxiety (*p* = 0.016). More severe depressive symptoms correlated to a more advanced stage of the disease. A multiple linear regression showed that disease stage and annual visits to the gynecologist are the risk factors associated with higher depression scores. *Conclusions*: Patients diagnosed with cervical cancer are a vulnerable group for the development of the psychiatric disorders and they require screening programs, which could potentially detect candidates for co-psychiatric and/or psychotherapeutic treatment. They demand particular attention because anxiety and depression are associated with the significant burden of the underlying disease and unfavorable survival rates.

## 1. Introduction

Globally, cervical cancer is the third most prevalent (9%) and the fourth most common cause of cancer mortality (8%) in women [1]. Cervical cancer is rarely detected in the early stages and can be easily neglected since it does not initially cause pain, and vaginal spotting is only sporadically reported. Nowadays, the incidence of cervical cancer is declining gradually, which can be explained by the early diagnostic tool application, the cervical Papanicolaou smear [2,3]. The known risk factors for developing cervical cancer are human papilloma virus (HPV), a low socio-economic status, smoking, marrying before the age of 18 years, young age at first coitus, multiple sexual partners, multiple sexual partners of spouse, and multiple childbirths. These factors raise the risk of developing cervical cancer [4]. The treatment of cervical cancer may consist of a combination of modalities such as surgery, chemotherapy, and radiotherapy [1]. Worldwide, cervical cancer is both the fourth main cause of cancer and a common cause of death in women [5].

Specific impairments in affect, perception, and social interaction are at the core of mental disorders. In the European Study of the Epidemiology of Mental Disorders, around 14% of the general European population report a lifetime history of a certain type of mood disorder, and 13.6% report anxiety during their lifetimes [6]. Probands with somatic diseases such as cancer are known to be at risk of concomitant psychiatric symptoms such as depression and anxiety, as well as a reduced quality of life (QoL). In the patients diagnosed with cancer, the prevalence of depression ranges from 8 to 24%. The percentage varies depending on the type of instrument used, the type of cancer, and the phase of treatment [7].

The incidence of mental disorders in patients with cancer is very high (30–60%) [8]. Earlier research in China also showed that 15.8% of cancer patients had clinical symptoms of a mental disorder, with depression, anxiety, psychotic symptoms, and stress-related disorders accounting for 13.3%, 10.2%, 2.8% and 1.4%, respectively [9]. Facing a life-threatening illness can cause anxiety, depression, and other psychological problems in many patients. Patients experience high levels of psychological stress, especially after diagnosis and at the start of treatment, which can place additional burdens on patients. Pain and fatigue, in particular, were negatively correlated with patient well-being and daily life [10,11]. Breast cancer, the most common malignant disease in women [12], is known to be closely related to psychiatric symptoms such as anxiety and/or depression, as well as impaired QoL. To the best of our knowledge, there are limited data regarding psychiatric symptoms and diseases in patients suffering from different genital cancers, which may also be a risk for the development of some psychiatric entities. Depression and anxiety are some of the most prevalent symptoms related to cancer, and their existence most likely influences treatment, compliance, and prognosis, since it affects the patient’s QoL [13,14,15].

This study aimed to explore and measure the level of anxiety and depression in cervical cancer patients, and to identify the possible predictors among known risk factors. In addition, this research might provide a helpful foundation for future research, as well as a better understanding of this vulnerable group, who might need more extensive psychosocial support during the course of their diseases.

## 2. Materials and Methods

### 2.1. Study Design

This research was designed as a prospective study and included 59 patients with cervical cancer who were treated at the Clinic for Oncology, University Clinical Center Nis; it is a part of a more extensive follow-up study. A consecutive sampling technique was used to select the participants. This method was chosen for this study due to the lack of a sampling frame for the study population. Patients were approached individually as they came into the clinic. They participated in the study after receiving a detailed description of the study aims. The disease is staged using the International Federation of Gynecology and Obstetrics (FIGO) system, and stages IIb, IIIa, and IIIb were considered advanced.

### 2.2. Participants

A total of 59 diagnosed (subsequently confirmed), willing, and physically able cervical cancer patients were enrolled in the study. Patients in FIGO stagse Ia, Ib, and IIa were initially subjected to surgery and, afterward, to the modalities of oncologic therapy. Due to the inability to differentiate therapeutic responses according to the type of therapy, they are excluded from this study. In addition, the patients in FIGO stage IV with metastatic diseases are also excluded. All examined patients with advanced cervical cancer (FIGO stages IIb, IIIa, and IIIb) had their diagnoses pathohistologically verified. Other exclusion criteria were: (I) age less than 18 years; (II) previous depressive disorders or other mental disorders preceding cervical cancer diagnosis; (III) inability to be followed up regularly, which was assessed by investigators based on the patients’ overall condition; (IV) human immunodeficiency virus infection; (V) presence of other malignant tumors; and (VI) pregnancy or lactation.

### 2.3. Assessement

A sociodemographic questionnaire and self-reported questionnaires measuring the levels of anxiety and depression were used in this research. Collected data included age, stage of carcinoma, use of barrier contraceptives, smoking status, time of initial sexual activity and number of sexual partners, as well as the frequency of gynecological examinations. All enrolled patients underwent an assessment for depression and anxiety using the Zung Anxiety Scale (SAS) [16] and Zung Depression Scale (SDS) [17] on the day they found out about their diagnosis. They were required to complete the SAS and SDS after instructions from a psychiatrist. The psychiatrist calculated the scores of the SAS and SDS. The subjects were divided into three groups according to their age and the stage of cancer.

The level of anxiety was measured by the 20-item Zung Anxiety Scale, based on scores obtained from 4 groups of manifestations: cognitive, autonomic, motor, and central nervous system symptoms. Each question is scored on a Likert-type scale from 1 (a little of the time) to 4 (most of the time). Some questions are negatively worded to avoid the problem of a set response. The overall assessment was made using a total raw score, which was converted to an “Anxiety Index”. The score groups were divided as follows: normal range (20–44), mild to moderate (45–59), marked to severe (60–74), and extreme (75 and above) anxiety levels.

The level of depression was measured by the 20-item Zung Depression Scale, a norm-referenced measure, used to screen adults for the potential presence of depressive symptoms; it is scored using a Likert-type scale. The total raw score was converted to an “SDS Index” and the score groups were divided as follows: normal (below 50), mild (50–59), moderate to marked major (60–69), and severe to extreme major depression.

### 2.4. Statistical Analysis

Analysis of the collected data was performed in SPSS version 20 (IBM Corp., Armonk, NY, USA). Among the basic descriptive statistical parameters, standard statistical methods were used for qualitative and quantitative assessments of the obtained results: absolute numbers, relative numbers (%), arithmetic mean ($\bar{X}$), and standard deviation (SD). The normality of the distribution was examined by the Kolmogorov–Smirnov test. A comparison of the arithmetic means of the two samples was performed using a *t*-test, while in cases of data not distributed normally, the nonparametric Mann–Whitney U test was used. ANOVA was used to compare the three samples. The χ^2^ test was used to test the statistical significance of absolute frequency differences between samples. Pearson’s correlation and Spearman’s rank correlation determined the correlation between the examined variables. Multivariate linear regression analysis was used to determine the predictors of depression and anxiety. The significance value of 0.05 or less was considered the significance threshold.

## 3. Results

The study included 59 patients who were in stages IIb, IIIa, and IIIb of the disease. The youngest patient included in the study was 41 years old, while the oldest was 75 years old, with a mean age of 58.02 ± 8.58 years. The patients were divided into three age groups: younger than 50 years (9 respondents), 51 to 60 years (30 respondents), and older than 61 years (20 respondents).

**Figure 1:**
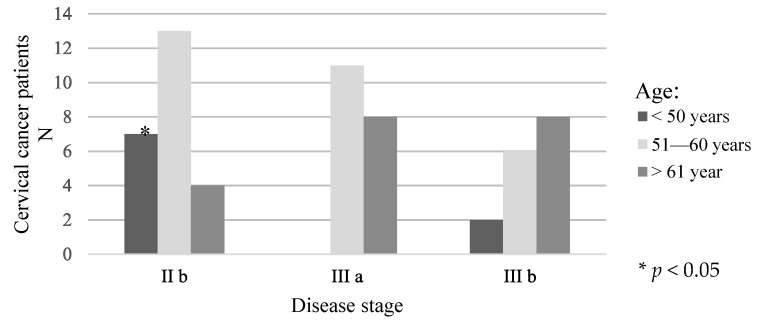
Distribution of patients in relation to age and disease stage.

The distribution of patients by age and stage of the disease is shown in Figure 1. Statistically significantly more often, respondents younger than 50 had stage IIb of the disease.

**Figure 2:**
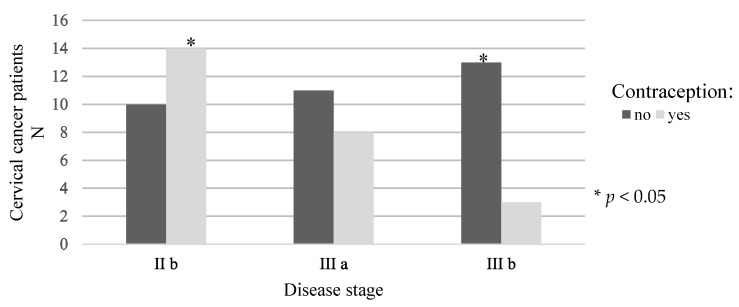
Distribution of patients in relation to the use of barrier contraceptives and disease stage.

The distribution of patients in relation to the use of barrier contraceptives and the stage of the disease is shown in Figure 2. Statistically significantly more subjects who did not use barrier contraceptives had stage IIIb disease, while statistically significantly more subjects who used contraception were in stage IIb of the disease.

No statistically significant difference was observed between respondents who had sexual intercourse before and after 18 years of age (*p* = 0.735), in relation to the stage of the disease. Additionally, no statistically significant difference was observed between respondents who had up to two and those who had more than two partners (*p* = 0.905). No statistically significant difference was observed between respondents who were smokers and those who were not in relation to disease stage (*p* = 0.309). Moreover, there were no significant differences between annual gynecological visits and disease stage (*p* = 0.437).

### 3.1. Prevalence and Severity of Depression and Anxiety among Cervical Cancer Patients, and Risk Factors

A significant correlation was found between disease stage and the scores of depression (χ^2^ = 16.570; *p* = 0.002) (Table 1) and anxiety (χ^2^ = 8.294; *p* = 0.016) (Table 2) in affected women. A significantly higher number of women with stage IIIb disease had scores that put them in the “severly depressed” group and anxiety scores that put them in the “mild to moderate anxiety” group.

### 3.2. Factors Affecting Depression Scores in Cervical Cancer Patients

The model of multiple linear regression analysis showed that disease stage (β = 0.473; *p* = 0.001) and annual visits to the gynecologist (β = 0.362; *p* = 0.006) were predictive factors for a higher depression score. (Table 3) The whole model was statistically significant (F = 3.978; *p* = 0.002) and explained 31.5% of the variance in depression scores.

### 3.3. Factors Affecting Anxiety Scores in Cervical Cancer Patients

The multiple linear regression analysis of parameters for the prediction of anxiety score in women affected by cervical cancer contained the following variables: age, disease stage, smoking status, number of partners, contraception, and annual visits to a gynecologist. The whole model is not statistically significant (F = 0.702; *p* = 0.649), so none of the examined variables contribute to the development of anxiety.

### 3.4. Correlation of Anxiety/Depression with Risk Factors in Cervical Cancer Patients

FIGO stage disease (*ρ* = 0.357; *p* = 0.006) and annual visits to gynecologist (*ρ* = 0.399; *p* = 0.002) had a positive correlation with depression scores. In the case of anxiety scores, no significant correlation was found (Table 4).

## 4. Discussion

Our results indicate that depression and anxiety scores are relatively high among the studied group of patients with cervical cancer, generally considered a vulnerable group. Severity levels of anxiety and depression differ amongst the studied groups and FIGO stages. The depression scores are higher in the group with the more advanced FIGO stage of the disease. Overall, the depression scores are significantly elevated in all three age groups. The FIGO stage and annual visits to a gynecologist are predictive factors for elevated depression scores in cervical cancer patients.

There are several possible explanations for the differences among the studied groups, since one might never be sure which mechanism underlies the changes in disease perception and data processing. The presence of cervical cancer and the associated fear of being sick might lead to an increased psychological burden, which could be responsible for the highly prevalent and aggravated anxiety/depression in patients when they receive a diagnosis. Additionally, impairment in everyday physical and social functions, the economic burden arising from treatment, and the psychological stress following the diagnosis itself—or even the anticipated results of different possible treatments—can also be associated with the high prevalence and severity of anxiety/depression in cervical cancer patients. In the end, one should not overlook the age difference between the patients.

Several previous studies reported similar findings [18,19,20], and it has been shown that anxiety and depression are associated with less favorable survival rates in cervical cancer patients [21]. On the other hand, a study reported lower values for the prevalence of depression, around 13%, in female patients diagnosed with genital carcinomas. The level of depression found in this study group is lower than the lifetime prevalence of depression in the general population, which is somewhere between 16% and 20% [22,23].

Sociodemographic characteristics are risk factors for anxiety and depression in patients who have cervical cancer [24]. Advanced tumor stage, financial difficulty (low income; potential unemployment), young age, lower education level, and overall low QoL can predict a high risk of developing depression and/or anxiety in cervical cancer patients [12,25,26]. Regarding sexual inactivity, studies have shown that, of 83 women, 61% were sexually active before the diagnosis, and almost half of the subjects reported a decline in sexual function after a cancer treatment protocol was conducted [19,25,27].

### Strengths and Limitations of the Study

There are several strengths and limitations of the current study. Firstly, patients with cervical cancer were recruited from only one department and, although it is a tertiary care facility, more subjects from different centers are needed in order to reduce selection bias. On the other hand, the nature of the tertiary care facility allowed a complete and detailed examination of the enrolled patients; thus, their selection would potentially be more adequate based on the inclusion/exclusion criteria. Secondly, self-reported scales used in this study have certain shortcomings, since the patients could minimize their mental issues and thus give false responses. One of the strengths is that this study is just a part of a larger study that provides basic information about the mental states of these vulnerable patients, and could be used to track future changes over the course of the disease.

## 5. Conclusions

This study assessed the levels of anxiety and depression and their relationship with known and established risk factors. FIGO stage and annual visits to a gynecologist were independently predictive factors for a higher risk of depression in women with cervical cancer. Patients diagnosed with cervical cancer are a group vulnerable to the development of the psychiatric disorders, and they require screening programs, which could potentially detect the candidates for co-psychiatric and/or psychotherapeutic treatment. They demand particular attention because anxiety and depression are associated with the significant burden of the underlying disease and unfavorable survival rates.

## Figures and Tables

**Table 1:** Risk factors and depression scores in cervical cancer patients.

	Mildly Depressed (n = 15)	Moderately Depressed (n = 40)	Severely Depressed (n = 4)	*p* Value
Age (years)	58.0 ± 9.6	57.6 ± 8.6	62.0 ± 4.3	0.631
FIGO stage, n (%)				0.002
IIb	8(53.3)	16(40.0)	0(0.0)	
IIIa	7(46.7)	12(30.0)	0(0.0)	
IIIb	0(0.0)	12(30.0)	4(100.0)	
Smoking, n (%)				0.471
No	7(46.7)	12(30.0)	1(25.0)	
Yes	8(53.3)	28(70.0)	3(75.0)	
Number of partners, n (%)				0.993
≤2	11(73.3)	29(72.5)	3(75.0)	
>2	4(26.7)	11(27.5)	1(25.0)	
Contraception, n (%)				0.490
No	10(66.7)	21(52.5)	3(75.0)	
Yes	5(33.3)	19(47.5)	1(25.0)	
Gynecological visits, n (%)				0.181
No	10(66.7)	17(42.5)	1(25.0)	
Yes	5(33.3)	23(57.5)	3(75.0)	

**Table 2:** Risk factors and anxiety scores in cervical cancer patients.

	Mild to Moderate Anxiety (n = 34)	Marked to Severe Anxiety (n = 25)	*p* Value
Age (years)	58.4 ± 8.0	57.6 ± 9.4	0.729
FIGO stage, n (%)			0.016
IIb	12(35.3)	12(48.0)	
IIIa	8(23.5)	11(44.0)	
IIIb	14(41.2)	2(8.0)	
Smoking, n (%)			0.050
No	8(23.5)	12(48.0)	
Yes	26(76.5)	13(52.0)	
Number of partners, n (%)			0.644
≤2	24(70.6)	19(76.0)	
>2	10(29.4)	6(24.0)	
Contraception, n (%)			0.752
No	15(44.1)	10(40.0)	
Yes	19(55.9)	15(60.0)	
Gynecological visits, n (%)			0.260
No	14(41.2)	14(56.0)	
Yes	20(58.8)	11(44.0)	

**Table 3:** The multivariate logistic regression model analyses of factors affecting depression among cervical cancer patients.

	Unstandardized Coefficient		Standardized Coefficient Beta	*p* Value
	B	SE		
Age	−0.011	0.072	−0.020	0.879
FIGO stage	2.715	0.743	0.473	0.001
Smoking	0.564	1.282	0.057	0.662
Number of partners	−0.121	1.336	−0.012	0.928
Contraception	0.215	1.302	0.023	0.870
Gynecological visit	3.377	1.190	0.362	0.006

**Table 4:** Correlation of depression/anxiety with risk factors in cervical cancer patients.

	Depression		Anxiety	
	r/*ρ*	*p* Value	r/*ρ*	*p* Value
Age	−0.143	0.279	−0.143	0.279
FIGO stage	0.357	0.006	−0.264	0.043
Smoking	0.230	0.079	−0.143	0.279
Number of partners	0.000	1.000	−0.016	0.905
Contraception	0.063	0.637	−0.036	0.789
Gynecological visits	0.399	0.002	−0.060	0.650

## Data Availability

Not applicable.
